# Children in South Africa with and without Intellectual Disabilities’ Rating of Their Frequency of Participation in Everyday Activities

**DOI:** 10.3390/ijerph17186702

**Published:** 2020-09-15

**Authors:** Alecia Samuels, Shakila Dada, Karin Van Niekerk, Patrik Arvidsson, Karina Huus

**Affiliations:** 1 Centre for Augmentative and Alternative Communication, University of Pretoria, Pretoria 0028, South Africa; shakila.dada@up.ac.za (S.D.); karin.vanniekerk@up.ac.za (K.V.N.); 2 CHILD Research Group, Swedish Institute for Disability Research, Jönköping University, 55111 Jönköping, Sweden; patrik.arvidsson@regiongavleborg.se (P.A.); karina.huus@ju.se (K.H.); 3 Centre for Research & Development, Uppsala University, Region Gävleborg, 801 88 Gävle, Sweden

**Keywords:** participation, attendance, children with intellectual disabilities, low- and middle-income country, self-report, Picture my Participation, activities of daily living

## Abstract

In a low-and middle-income country (LMIC) such as South Africa, not much is known about how children with intellectual disabilities (ID) participate in everyday activities, as no studies to date have compared their participation to peers without ID from the same background. Using a newly developed, contextually valid measure of participation, Picture my Participation (PmP), 106 children with (73) and without ID (33), rated their frequency of participation in activities of daily living. Previous international research has established that children with ID tend to participate less frequently than children without ID in everyday activities outside of the school setting. However, much of this research is based on proxy ratings from caregivers rather than children with ID themselves. There is a growing body of evidence that suggests children with disabilities have uniquely different views of their own participation than their caregivers. The existing research evidence is also delimited to studies conducted predominantly in high income contexts (HICSs). Since it is universally acknowledged that participation patterns are affected by the environment, it is important to evaluate the generalizability of the current evidence to LMICs. The current study found that there were many similar patterns of participation between the two groups although significant differences were noted in social, community, leisure and self-care activities. We compare these results to findings from studies conducted in HICs and find that there are similarities but also differences across contexts. This study highlights the importance of gaining a child’s perspective of participation and understanding how intellectual disability can affect participation relative to peers without ID in LMICS.

## 1. Introduction

The WHOs International Classification of Functioning, Disability and Health (ICF) [1], more specifically its version for children and youth (ICF-CY) [2] is a framework that seeks to provide an understanding of children’s functioning. It has brought about a change in the way we view outcomes for children with intellectual disability (ID) with the concept of participation increasingly seen as an important goal for this population. Participation is defined in the ICF-CY as a child’s “involvement in a life situation” which can be influenced by factors such as child characteristics (health conditions, body functions, and structures) and context (facilitators or barriers of the physical or social environment) [2].

King and colleagues [3] view participation in everyday activities as the context in which children develop competencies, where they gain an understanding of their strengths and abilities, form friendships and relationships, and ultimately make a contribution to their worlds. Participation is therefore both the ultimate goal of intervention and the context in which children develop [4].

The participation construct is especially important for children with ID since behavioral problems and social skills deficits tend to restrict their participation in most of their everyday environments such as home, school and community [5]. Some studies have suggested that many children with disabilities participate in less diverse activities as well as less frequently in activities than their typical peers [3]. Participation can contribute amongst others, to children with ID’s health, self-esteem and psychological well-being [5]. Specifically, they may be at risk for increased sedentary lifestyles and social isolation [3].

While the ICF defines participation as “involvement in a life situation”, this definition has been criticized by various authors as being difficult to operationalize and measure [6,7]. This difficulty stems from the failure of the ICF as a classification system to adequately differentiate the activity competence domain (measured as capacity, capability or performed skill in being able to execute an activity) from that of participation [8,9]. Consequently, many studies which claim to measure participation in childhood disability research tend to measure a broad range of constructs related to participation such as activity competence, preference, or aspects related to sense of self [9,10,11], rather than the social aspects of participation.

Granlund [6] and colleagues [12], based on a critical review of the language of participation used in the literature, recommend that participation be differentiated into the dimensions of “being there” (measured as time spent in or frequency of attendance in a life situation) and involvement (the experience of participation while attending in a life situation also referred to as engagement). Apart from a few exceptions, a systematic review of the literature on the participation of children with disabilities [5] revealed a tendency for the “being there” dimension of participation to be measured more often (i.e., frequency of access and inclusion in various activities of daily living) than involvement or engagement in these activities [13].

With specific reference to children and youth with ID, Shields and colleagues’ [5] systematic review found that that when socio economic status is controlled, there are more similarities than differences in participation patterns pertaining to outside school activities between children with ID than peers without ID. Of the four studies that met the inclusion criteria for their review, children with ID were found to participate in a similar number of activities and to the same extent in terms of frequency for leisure activities (reading, watching television, computer games, listening to music) and physical community activities (e.g., sporting activities, biking, taking music or dance lessons). Both groups also participated with the same frequency in more informal, outdoor and accessible activities. Notable differences were reported in relation to the decreased number of social community activities and recreational activities participated in by children with ID, as well community activities, family enrichment activities and formal activities. Frequency of participation was different in relation to organized physical activities, playground activities with siblings, errands, dining out, as well as community activities such as visiting the library, or going to the movies. In contrast, children with ID tended to participate in a higher percentage—although less frequently—in community based social activities with their parents and other adults than with similar aged peers. According to Shields and colleagues [5] if the usual patterns of participation of children with ID are known in comparison to their peers without ID, it could potentially help us to understand whether or not they have opportunities to pursue activities that they enjoy which would contribute to their health and development.

The current systematic synthesis of literature on participation of children with ID, however, suffers from two biases found when measuring children with disabilities’ participation. Firstly, much of the findings of the existing literature are based on proxy ratings from caregivers [10,14] Caregiver perspectives of participation may be very different to children’s own self-reported experiences [3] as attested to in a recent study by Huus and colleagues [15] on perceptions of children with ID’s human rights. Larger differences were found when children as self-raters were asked about whether they had things or friends to play with at home compared to their parent’s proxy rating of this same question. This study also revealed that child-parent agreement can be affected by socio economic variables.

Secondly, the evidence base is currently delimited to studies conducted in high income contexts [16]. The ICF, based as it is on an ecological model of childhood development and a biopsychosocial perspective, acknowledges the situational nature of participation with the environment viewed as a key influencing factor [17]. In LMICs where resource limitations and negative attitudes towards people with disabilities are more acute [18], children with ID may be more restricted in their participation than their peers in high income contexts. It has been established that environmental factors such as cultural context [9] and socio-economic status [5] are related to children with disabilities’ frequency of participation in different everyday activities. Both of these limitations on the current research evidence accentuate the importance of including children’s own voices in perceptions of their participation in LMICs.

A recent systematic review focused on examining the participation construct in LMICs [16] revealed no studies to date in LMIC’s where children with ID have evaluated their frequency of participation in various out of school environments. The current study thus sought to evaluate children with ID’s ratings of their frequency of participation in various every-day contexts compared to their peers without intellectual disability in South Africa. Furthermore, since current self-report instruments of children’s participation may not be contextually valid for use in LMICs, a new self-report measure for children with disabilities, Picture my Participation (PmP) [19], that takes into account culturally relevant activities that may be more relevant in LMICs, was used. More detail about the development and preliminary validation of the PmP in LMICs has been published elsewhere [19] and more detail about how it was used in this study is provided in the methodology of this paper. However, as one of the few self-report measures of participation that provides a child’s perspective [20] it may provide a more valid understanding of the participation patterns of children with ID as well as the everyday activities they deem important [20]. While children as young as 4 to 5 years of age are able to reliably complete self-report measures if items and rating scales are suitably adapted [10]. Special consideration should be given to adapting measures for children with disabilities especially those with cognitive and communication impairments [20,21]. Measures for example that are cognitively accessible [21], i.e., that take a range of cognitive abilities into account and provide supports to reduce cognitive demands, increase the chances that respondents can interpret and respond to items as they were intended. The PmP provides visual supports using visual symbols for activity items and participation rating scales and takes the form of an interview with children with disabilities. These accessibility features thus contribute to more valid and reliable self-reporting [21].

## 2. Materials and Methods

### 2.1. Ethics

Ethical approval for the study was obtained from the University of Pretoria’s Faculty of Humanities Research Ethics Committee (GW20180301HS) on the 10th of April 2018 and permission to conduct interviews was obtained from the local Department of Education and school principals. Informed consent was obtained from every child’s primary caregiver, and assent was also sought from each participating child.

### 2.2. Recruitment

Dyads of caregivers and children were purposefully selected to participate in the study. All caregivers were required to be English literate, as they were required to complete the survey in English. Furthermore, the caregivers were required to be the primary caregiver of the child (that had to meet the criteria set for the children).

Children with ID were recruited from two special schools in Pretoria, South Africa that had English as the medium of instruction for teaching and learning. Up to 70% of children with disabilities in South Africa are not in school and when they do attend, most are still in separate schools for children with disabilities, termed special schools [22]. This situation continues despite progressive policies that are required to promote inclusive education. Children in these special schools come from diverse cultural and socioeconomic backgrounds.

In order to participate in the study, the children were required to (i) be older than 10 years and younger than 16 years. They were (ii) required to have attended a school with English as language of teaching and learning for a minimum of 2 years, in order ensure their ability to comprehend the instructions of the Picture my Participation tool. The participants were required to be (iii) diagnosed as having mild to moderate intellectual disability in order to be included in the group of children with disabilities. Furthermore, the children with ID were required to (iv) not have any functional hearing, visual or motor impairments.

The children without ID were recruited from mainstream schools in Pretoria, Gauteng, that all had English as the medium of teaching and learning. While in principle a mainstream school in South Africa refers to a neighborhood school that all children regardless of ability should be able to attend; in reality, however, it tends to cater to children who do not have special education needs [22]. All the children without ID were (i) between 10 years and 15 years and 11 months; (ii) had attended their current school for a minimum of 2 years and (iii) had never failed a grade at school. All recruited children were from an urban environment. Descriptive statistics regarding the participants can be viewed in Table 1.

### 2.3. Instruments

In order to determine children’s eligibility for inclusion into the study, the following measures were completed with each child before the onset of conducting PmP interview. The Kaufman Brief Intelligence Test Second Edition (KBIT-2) [23] is a test of intelligence that is often used in research as a screening tool that can be administered by non-psychologists. Clinical researchers and specially trained postgraduate students with knowledge of the target group, completed this measure with children.

The Ten Questions Questionnaire (TQQ) [24] was completed by children’s primary caregivers to rule out any hearing, vision or motor impairments. This is a screening tool that assesses the level and nature of a child’s disability and includes 10 closed (yes/no) questions about whether the child has a problem or not in certain developmental areas.

In addition, the Learner Screening Tool by Educators (LeSTE) [25] was completed by teachers and the Line Drawings Test [26] was completed by a member of the research team with the participants. These tests provided additional information about the sensory and motor abilities of each child.

The PmP instrument was developed as a self-reported measure of participation of children in home, social and community activities within LMICs [19]. The PmP has two sections—Section 1 is completed by parents and Section 2 is a self-reported measure completed by an interviewer according to responses received from a child [19]. Section 1 starts with a consent form for the parent and is followed by demographic questions as well as the Ten Questions Questionnaire [24]. If the child meets the inclusion criteria, the parent will also complete the rest of the survey that contains the same questions pertaining to the frequency of the child’s attendance and their perceived involvement in 20 activities as those included in the questions to the children.

Section 2 includes an assent form that is completed with a child before the commencement of the interview. Thereafter, children are asked to rate the frequency of their attendance in 20 different activities (developed from the ICF) according to a four-point symbol-based, visual Likert scale using Picture Communication Symbols (PCS). All 20 activities were also represented by a PCS symbol of the activity. The scale was also presented to the children visually by four PCS symbols that were displayed on a mat and kept in front of the child during the interview.

The symbols on the mat depicting the scale were of baskets with apples, filled to various degrees. “Always” on the scale was represented by a basket completely filled with apples, “sometimes” by a basket with three apples in it, “not really” by a basket with one apple and “never” by an empty basket. The Talking Mats methodology as described by Cameron and Murphy [27] was used and enabled the children to place the symbol of the activity that they were being asked about, under the appropriate visual Likert scale symbol displayed on the mat. The children were asked three trial questions to ensure that they understood the scale.

The children were also asked about their level of involvement in the 20 activities which they had to rate on a scale with “very”, “somewhat” and ”minimally” also represented in symbols on the mat. Thereafter, the children were required to state three activities that were most important to them. Following this, they were asked to indicate their perceived barriers and facilitators to participation for these three activities. In this paper, only the data on the frequency of attendance obtained from the children themselves will be analyzed and discussed. An interview with a child took approximately 30 min to complete.

### 2.4. Data Analysis

Statistical analysis was performed using SPSS software (version 25) (IBM Corp, Armonk, NY, USA). Since the data were not normally distributed, differences in participation in terms of frequency of attendance for each activity item on the PmP between groups of children with and without ID were analyzed using the non-parametric Mann–Whitney U test. This statistical test compares mean rank data and not the means as is typically done when the data are normally distributed.

*P*-values < 0.05 were considered as significant. Spearman’s rank-order correlation was used to explore the relationship between the total scores of participation (a summary of frequencies of attendance for all activity items) between children with and children without disabilities.

## 3. Results

The results of a Mann–Whitney U test indicated several significant differences between the children with and without intellectual disabilities for 9 out of 20 items of the PmP (Table 2).

Firstly, children with ID indicated that they participated significantly less than TD children in several social activities and taking care of others. These activities included meal preparation: with or for the family (*p* < 0.001 *), spiritual activities: religious and spiritual gatherings and activities (*p* < 0.001*) as well as social activities: taking part in social activities in the community (*p* = 0.006 *). Furthermore, children with ID participated significantly less than TD children in several family life activities. These include family time: interact with the family (*p* < 0.001 *); family mealtime: (with usual family members) (*p* = 0.001 *); as well as quiet leisure (listening to music, reading) (*p* = 0.003 *).

Finally, several significant differences were observed between children with ID and children with typical development as the children with ID participated significantly less in personal care and development activities. These include school: formal learning at school (*p*< 0.001 *), personal care: daily routines at home for personal care (*p* = 0.048 *) as well as looking after their own health (*p* = 0.001 *).

A Spearman’s rank-order correlation determined the relationship between the total scores, i.e., the summary of frequencies for every item, between children with and children without disabilities. There was a strong, positive correlation between children with and without disabilities, which was statistically significant (rs = 0.868, *p* < 0.001).

## 4. Discussion

Since it has been well established that factors within and outside of the child can act as barriers or facilitators of participation [2], we compare the findings of the current study to comparable studies undertaken in HICs on children with and without IDs frequency of attending various everyday activities. Where unexpected findings are noted, we cautiously infer from contextually relevant literature as to possible reasons that could account for differences. However, we are mindful that apart from obtaining demographic data such as economic status, we did not specifically measure how child characteristics or environmental factors could have accounted for the results.

In this study, the finding that children with and without ID from comparable socioeconomic backgrounds, display more similarities than differences in their frequency of attending everyday activities appears to be a consistent trend across low- and high-income contexts [5]. In South Africa, children with ID participated to the same extent in terms of frequency of attendance as children without ID in the majority of activities measured by the PmP. However, while there may be more similarities than differences between children with and without ID in terms of frequency of attendance across economic contexts, the actual activities where children with ID appear to have similar frequencies of attendance to their peers, tend to be different across economic contexts. In South Africa, similar patterns of participation to peers without ID are for activities taking place within the home environment and within the context of family routines such as household chores, taking care of family members, caring for pets and assisting with gathering basic necessities for the family. This may be a function of receiving more support in the home environment from family members than in outside contexts where they may not be present [19]. This is supported by King and colleagues [3] who found that children with ID had a higher frequency of participation in social activities in the home. This may be indicative of more support being offered in these environments from family members due to reduced physical, cognitive and social skills associated with this population [3].

In HICs, similar patterns of participation in terms of frequency of attendance between children with and without ID was noted in mainly leisure and community activities such as attending play groups, childcare and church [5]. These differences in the type of activities that were reported to have similar frequencies of participation between children with and without ID may be as a result of using different measuring instruments and therefore a function of content validity of instruments. In developing the PmP for LMICs, Arvidsson and colleagues [19] note the importance of having everyday activity items that are culturally relevant, i.e., meaningful and important for the population in a specific context.

However, there were also quite a few significant differences between children with and without ID, and these will be discussed in more detail in relation to specific activity types.

### 4.1. Social (Iutside the Home) and Community Life Activties

As noted above, while the present study found that there were many social activities in the home where children with ID participated similarly to peers without ID, notable differences included social activities outside the home, such as attending parties, playgroups or parades as well as community activities such as attending religious or spiritual gatherings. Findings of decreased frequency of participation for children with ID relative to peers without ID in social activities outside the home are similar to studies conducted in Australia [28] although in Canada, King and colleagues [3] found no differences between the two groups. Further investigation is therefore warranted into some of the potential barriers which may be unique to children with ID in South Africa and which could potentially explain the differences noted. Although we did not specifically test for any predictive or causative factors that would influence frequency of participation in out of home social and community activities, the South African literature alludes to potential physical access barriers in the community faced by people with ID, such as a lack of reliable, affordable and accessible public transport infrastructure [29] that could potentially limit children with ID’s ability to participate in activities outside of the home.

As noted, participation can also be influenced by child characteristics such as an impairment in body structures and functions [2]. Children with ID in South Africa tend to have associated communication impairments in terms of receptive and expressive deficits [30,31] which can potentially limit their ability to independently access understandable information in their social environments [29], e.g., transport timetables and schedules. This may also be compounded by the fact that the special school in system South Africa rarely prioritizes literacy outcomes [32]. Thus, many children with ID exit the education system as non-literate, which can influence their ability to access knowledge and some degree of independence in social situations [29]. Communication difficulties may also limit children with IDs communicative interactions [30] in social situations such as parties or celebrations but their poor participation in social activities could also be affected by their associated deficits in social skills [5].

The significant differences between children with and without ID in terms of attendance of community activities such as religious or spiritual festivities is not supported by data from HICs, where children with and without ID display little difference when participating in this community activity [5]. The differences noted in the South African context may be a feature of the environment, such as attitudinal barriers towards people with intellectual disability [33].

Ndlovu argues that in some (although not all) indigenous African religions of Southern Africa, individuals with psychological and neurological impairments are often depicted as victims of witchcraft who should be “ritually, morally and physically cleansed of their affliction before they can be reintegrated into human society” [33] (p.32). For children with ID who have a lifelong health condition, these attitudinal barriers that equate the origins of ID with misfortune from the spiritual world, mitigate against inclusive practices and can result in continued marginalization and exclusion in religious and community life [34]. Again, we can only infer from the literature as to potential influencing factors. This will need to be empirically tested using study designs which specifically explore the impact of child characteristics and environmental factors such as physical, social and attitudinal barriers [35] on out of home social and community participation of children with ID.

### 4.2. Participation in Family Life and Indoor Leisure Activities

Differences in participation in family life and leisure activities in the home were also noted between children with and without ID in the current study. Children with ID indicated that they participated less frequently than children without ID in spending time with usual family members or interacting with the family members during mealtimes. This finding is somewhat inconsistent with studies conducted in Australia [28] where children with ID participated to the same extent as peers without ID in recreational activities with family members such as parents or with another adult: It is believed that family members are important forms of support for children with ID in situations which place a load on their cognitive and linguistic abilities [5]. However, this finding may reflect a preference for activities that are easier to engage in and for which they may not need as much support having developed the skills to participate independently [36].

This study also indicated that children with ID participated less frequently in quiet leisure time activities—such as listening to music or reading—than peers without ID. This is similar to results reported by Axelsson and Wilder [37] within the Swedish context, albeit with a more profound ID population. Less frequent participation in quiet leisure activities such as reading is also consistent with previous research [5], but as previously mentioned, poor prioritization of literacy outcomes for children with ID in South Africa [32] may account for a lack of participation in this activity.

Children with ID also indicated that they participated less frequently in their own personal care routines (dressing, brushing teeth, etc.) looking after their own health and development enhancing activities such as formal learning at school in comparison to their peers without ID. Due to the physical and cognitive demands associated with these activities, it may be a reflection that they may not have developed the functional skills needed to participate independently in these activities [3]. There is a strong body of evidence to suggest that children with ID in the South African context, like children with ID elsewhere, are at increased risk for oral health disease such as gingivitis and dental caries relative to the general population of similar age [38]. It is argued that the ability to maintain optimum oral health independently is difficult for this population and that support from caregivers for daily self-care activities and health remains high [39]. On the other hand, it has also been suggested that in some cultures, mothers’ strong commitment to their caregiving role may impact on children with ID’s ability to do personal care tasks for themselves [40]. More specifically, it has been argued that parents in collectivist cultures’ strong values of care and protection may limit the ability of their children with IDs to develop functional independence in activities such as personal care routines [40]. However, the evidence for this argument is limited and would need to be investigated in much more depth for it to be seen as a significant barrier limiting children with IDs participation in self-care and health related activities.

Despite this study’s findings of some significant differences in the frequency of participation in everyday activities between children with and without ID in the South African context, there is a growing realization in the country of the importance of participation and independence for children with ID in leisure, family and community life [41]. Some useful community-based rehabilitation interventions [42] that target the empowerment of the caregivers of children with disabilities to be advocates for their children, are starting to break down environmental barriers to their community participation. This is important as the decreased participation of children with ID from social, community and leisure activities, may have considerable implications for their social skills development and network of friendships within their peer cohort which are important for their quality of life and well-being [5].

## 5. Conclusions

This study, which compared the frequency of participation in everyday activities between children with and without ID from their own perspective using a contextually valid, self-report measure, is one of the first studies in South Africa to evaluate the similarities and differences in participation patterns for this population. While many similarities to studies undertaken in HICs were found, there were also some significant differences reported. At this stage, we were only able to infer from the literature as to possible reasons for this, as we did not measure any environmental factors or child characteristics which could account for the similarities and differences noted. We acknowledge this as an important limitation of the study which would need to be addressed in future research.

While children with ID participated similarly to peers without ID in the majority of activities in terms of frequency of attendance, it does not necessarily mean that they were involved in the activities to the same extent. This is an aspect which future research would need to address as well, since there are still very few studies—particularly in LMICs—which focus on the involvement dimension of participation [16]. Ultimately the knowledge of how children with ID participate in everyday activities will allow clinicians and researchers to gain better insight of how and where to target interventions to improve children with IDs’ participation in everyday activities and which will ultimately contribute to their health and development [5].

This study, however, is one of the first in LMICs to evaluate and compare the participation of children with ID to their peers without ID in terms of frequency of attendance in activities from the perspective of children themselves. New participation tools, such as the PmP—which are more cognitively accessible for children with disabilities and take contextually relevant activities into account—represent an important step for increasing research knowledge about how children with ID participate in LMICs. Ultimately, this broadens the knowledge base and the external validity of participation research.

## Figures and Tables

**Table 1:** Descriptive information on the participants.

Demographic Variables	Children without ID	Children with ID
	*n* = 33	*n* = 79
Gender		
Male	11 (33.3%)	38(48.1%)
Female	22 (66.7%)	36 (45.6%)
Missing	0	5 (6.3%)
Age		
9 years	3 (9.1%)	2 (2.5%)
10 years	16 (48.5%)	5 (6.3%)
11 years		13 (16.5%)
12 years	1 (3.0%)	16 (20.3%)
13 years	12 (36.4%)	14 (17.7%)
14 years	1 (3%)	11 (13.9%)
15 years	0	11 (13.9%)
16 years	0	2 (2.5%)
Missing	0	5 (6.3%)
Total family income		
Below R6000 per month	23 (69.7%)	55 (69.6%)
Above R6000 per month	7 (21.2%)	9 (11.4%)
Missing	3 (9.1%)	15 (19.0%)
Relationship of caregiver to child		
Father	7 (21.2%)	10 (12.7%)
Mother	25 (75.8%)	52 (65.8%)
Grandmother	0	1 (1.3%)
Other	1 (3.0%)	6 (7.6%)
Missing	0	10 (12.7%)
Employment status of caregiver		
Employed full-time	21 (63.6%)	35 (44.3%)
Part time	3 (9.1%)	12 (15.2%)
Unemployed	9 (27.3%)	22 (27.8%)
Missing	0	10 (12.7%)
Receiving social grant		
Yes	1 (3.0%)	26 (32.9%)
No	32 (97.05)	44 (55.7%)
Missing	0	9 (11.4%)
Highest education level of caregiver		
Grade 10 or less	1 (3.0%)	19 (24.1%)
Grade 12	9 (27.3%)	17 (21.5%)
Diploma	12 (36.4%)	10 (12.7%)
Degree	4 (12.1%)	5 (6.3%)
Postgraduate degree	1 (3.0%)	7 (8.9%)
Missing	6 (18.2%)	21 (26.6%)

**Table 2:** Differences in participation (frequency of attendance) of each activity item between children with ID (*n* = 73) and children with typical development (TD) (*n* = 33).

PmP Items		Mean Rank	Mann–Whitney U	Z	Exact Sig. (2-taile)]
Personal care: Daily routines at home for personal care (dressing, choosing clothing, hair care, brushing teeth)	TD child	60.35	978.500	−1.982	0.048 *
	Child with ID	50.40			
Family mealtime: (with usual family members)	TD child	66.05	790.500	−3.242	0.001 *
	Child with ID	47.83			
Looking after his/her own health	TD child	67.50	742.500	−3.263	0.001 *
	Child with ID	47.17			
Gathering daily necessities for the family (water, food, picking vegetables, fuel)	TD child	50.05	1090.500	−0.812	0.419
	Child with ID	55.06			
Meal preparation with or for the family	TD child	68.75	632.000	−3.822	0.000 *
	Child with ID	45.28			
Cleaning at home: Cleaning up at home [clothing, household objects, laundry, rubbish, yard work	TD child	60.47	974.500	−1.683	0.092
	Child with ID	50.35			
Caring for family: Taking care of other family members	TD child	56.27	1113.000	−0.659	0.510
	Child with ID	52.25			
Caring for animals/pets: Taking care of animals (pet, or domestic livestock)	TD child	55.58	1136.000	−0.489	0.633
	Child with ID	52.56			
Family time: Interact with the family	TD child	69.92	662.500	−4.129	0.000 *
	Child with ID	46.08			
Celebrations: Family/community celebrations (birthdays, Weddings, Holiday gatherings)	TD child	60.30	980.000	−1.632	0.108
	Child with ID	50.42			
Playing with others: Getting together with other children in the community	TD child	58.06	1054.000	−1.083	0.280
	Child with ID	51.44			
Organised leisure: sport, clubs, music, art, dance	TD child	57.23	1081.500	−0.875	0.381
	Child with ID	51.82			
Quiet leisure (listening to music, reading)	TD child	65.59	805.500	−2.921	0.003 *
	Child with ID	48.03			
Religious and spiritual gatherings and activities	TD child	69.56	674.500	−3.900	0.000 *
	Child with ID	46.24			
Shopping and errands (market)	TD child	58.77	1030.500	−1.254	0.210
	Child with ID	51.12			
Social activities: Taking part in social activities in the community (parties, play group, parades)	TD child	64.30	815.000	−2.738	0.006 *
	Child with ID	47.82			
Visit to health centre (e.g., Doctor, dentist, other health care service)	TD child	53.05	1189.500	−0.109	0.949
	Child with ID	53.71			
School: Formal learning at school	TD child	68.53	708.500	−4.161	0.000 *
	Child with ID	46.71			
Overnight visits and trips	TD child	60.35	978.500	−1.634	0.104
	Child with ID	50.40			
Paid and unpaid employment	TD child	47.12	994.000	−1.520	0.125
	Child with ID	55.69			

* p < 0.05.
